# Comparison of different embolic particles for superior rectal arterial embolization of chronic hemorrhoidal bleeding: gelfoam versus microparticle

**DOI:** 10.1186/s12876-021-02046-3

**Published:** 2021-12-14

**Authors:** Xuemin Wang, Yuguo Sheng, Zhu Wang, Wenming Wang, Fengfei Xia, Mengpeng Zhao, Xinqiang Han

**Affiliations:** 1 Department of Gastroenterology, Binzhou Medical University Hospital, Binzhou, Shandong People’s Republic of China 256603; 2 Department of Interventional Medicine and Vascular, Binzhou Medical University Hospital, No. 661 Huanghe 2nd Road, Binzhou, 256603 Shandong Province People’s Republic of China; 3 Department of Interventional Medicine and Vascular, Binzhou Peoples Hospital, Binzhou, Shandong People’s Republic of China 256600

**Keywords:** Hemorrhoids, Superior rectal artery, Embolization, Interventional, Emborrhoid

## Abstract

**Background:**

Whether different embolic particles with comparable diameter lead to similar beneficial effects in endovascular embolization of hemorrhoidal disease remains to be established. We sought to evaluate the efficacy and safety of different types of agents for superior rectal arterial embolization (SRAE) in patients with bleeding hemorrhoids.

**Methods:**

Patients with recurrent episodes of internal hemorrhoidal bleeding and chronic anemia treated by SRAE in three tertiary hospitals between March 2017 and June 2020 were retrospectively evaluated. The patients were divided into two study groups based on the embolic materials: embolization with coils (2–3 mm) + gelfoam particles at 350–560 μm (Group A, n = 23), embolization with coils (2–3 mm) + microparticles at 300–500 μm (Group B, n = 18). The technical success, preliminary clinical efficacy (percentage of patients without hematochezia), postoperative complications and short-term follow-up outcomes were analysed.

**Results:**

A total of 41 patients (27 males) with symptomatic hemorrhoids were included in the study, mean age was 47 ± 12 years (range 25–72). 39% (16) patients with grade II hemorrhoids while 61% (25) patients with grade III. The technical success rate of the embolization procedure was 100%, and the preliminary clinical efficacy (87.0% vs 88.9%) showed no significant difference between the 2 groups (*p* = 0.098). No patients reported post-procedural and short-term serious complications, such as infection, intestinal ischemia or massive hemorrhage during the follow-up period (range 6–15 months).

**Conclusions:**

Both gelfoam particles and microparticles with comparable diameter in the endovascular treatment of hemorrhoidal bleeding demonstrated similarly good short-term efficacy and safety profile.

## Background

Hemorrhoidal disease (HD) is one of the most common benign anorectal disease. Hematochezia is the most prevalent clinical manifestations causing anemia in the long-term and with a significant deterioration of the patients’ quality of life [1]. The treatment of HD has dramatically evolved over recent years.

In 1995, the Doppler-guided haemorrhoid artery ligation (DG-HAL) technique was first used by Morinaga to treat grade II–III internal hemorrhoids [2]. DG-HAL reduces the arterial inflow of internal hemorrhoids by ligating the superior hemorrhoidal artery (SRA), which can achieve 90% hemostasis efficiency. Some recently conducted studies on cadavers have shown the arterial blood supply of the corpus cavernosum recti (CCR) and the characteristics and distribution of the different rectal arteries [3, 4]. The findings underscore the role of the SRA terminal branches as primary blood supply of the hemorrhoidal cushions [3]. The rapid development of interventional devices and embolization techniques provides a new method for the therapy of vascular diseases that were previously managed using more aggressive surgical approach. On this basis, Vidal had attempted this endovascular embolization of the SRA in 2014, procedure known as “emborrhoid” and which is itself a technique based on pathophysiological characteristics of arteriovenous network hypertrophy in HD and transanal dearterialization and ligation of the hemorrhoidal arteries [5], and the preliminary findings are encouraging [6]. Additionally, the procedure with less trauma, significantly less pain during intraoperative and postoperative, and faster postoperative recovery. Therefore, the “emborrhoid” technique has been proven as a good alternative treatment method for internal hemorrhoidal bleeding, especially for patients with surgical contraindications or refusing conventional hemorrhoidectomy [1, 6–10].

However, there has been no consensus regarding which embolic material is best in the endovascular treatment of hemorrhoidal bleeding [7, 11–13]. Therefore, our study aimed to compare the results of different embolic particles for SRA embolization (SRAE) in treating grade II–III internal hemorrhoidal bleeding in a retrospective cohort of patients with no prior surgical history.

## Methods

### Patients

Fifty-four consecutive inpatients with symptomatic hemorrhoids of Goligher grades [14] II–III, who underwent SRAE for chronic hemorrhoidal bleeding in three tertiary hospitals from March 2017 to June 2020 were retrospectively evaluated.

Of these patients, forty-one patients were selected for the study by exclusion criteria. The exclusion criteria were the following: (i) embolization by more than two embolic agents or other than gelfoam and microparticle; (ii) embolic particles with different diameters; (iii) lost to follow up during 6–15 months after discharge; (iv) previous history of SRAE or hemorrhoidal surgery. The patients were divided into two groups based on the embolic particles: Group A (coils + gelfoam particles) and Group B (coils + microparticles), and the types of particles used in each patient was randomly selected.

Hematochezia was the predominant symptom and the severity of the bleeding was similar at baseline in all patients according to the French bleeding severity score (“Appendix”) [15]. All patients, regardless of age, were suffering from persistent symptoms despite conservative measures that including dietary or lifestyle adjustment and topical or oral medication. And some of them had obvious surgical contraindications such as poor cardiopulmonary reserve and disorders of blood coagulation, and some refused conventional hemorrhoidectomy.

### Angiography and embolization procedure

Preoperative examinations were performed (digital rectal examination, colonoscopy, complete blood count and coagulation tests) to exclud malignant tumor or other sources of bleeding. This treatment process was planned by a multidisciplinary team consisting of interventional radiologist, proctologist and gastroenterologist. Prior to the procedure, all patients provided written informed consent.

**Fig. 1:**
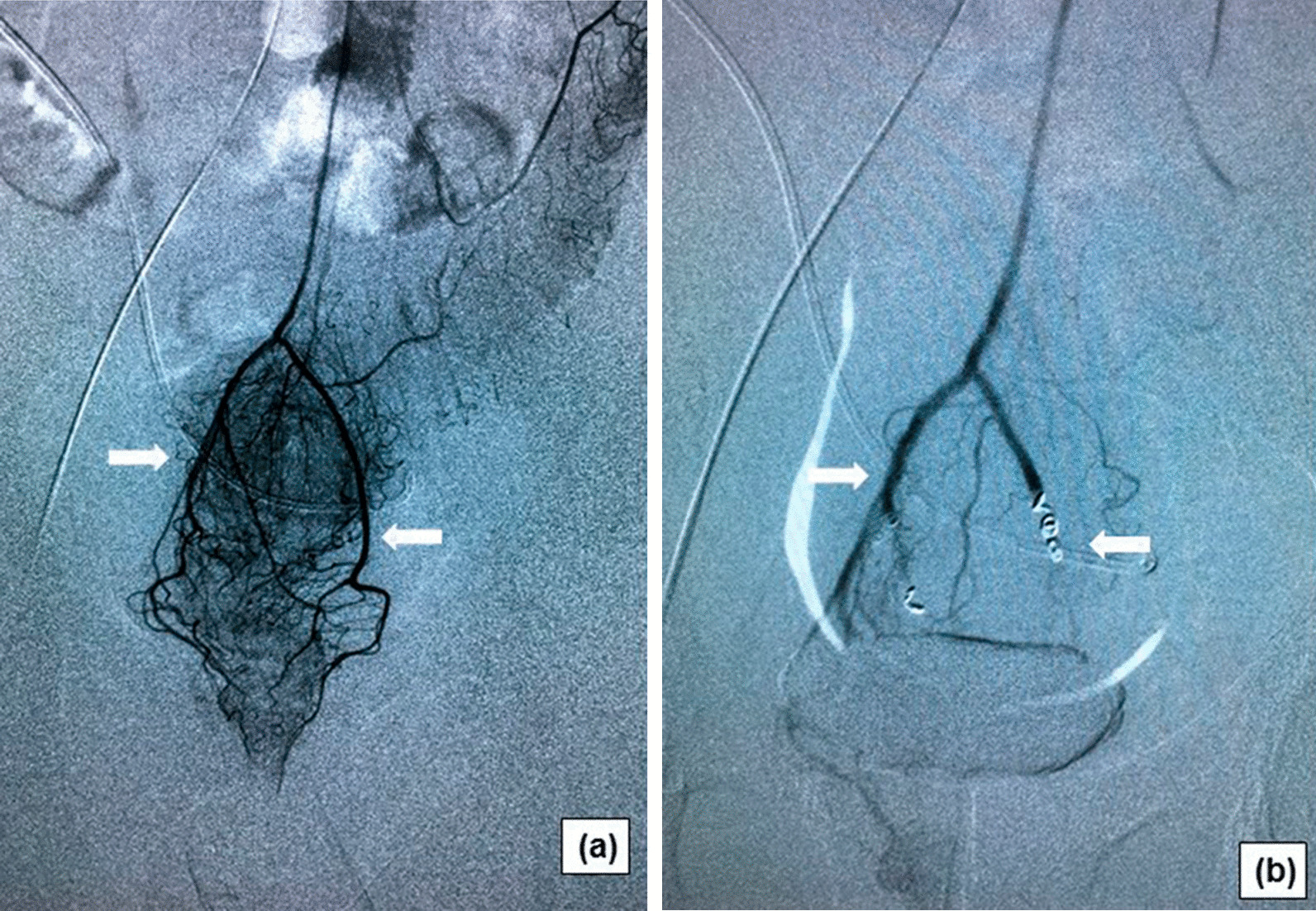
Angiographic features of hemorrhoid artery in patients with internal hemorrhoids. **a** Catheterization of the inferior mesenteric artery with a Simmons catheter showing the rectal hypervascularization (white arrows); **b** The final arteriogram showing reduced vascularization of the inferior part of the rectum after embolization of both SRAs using coils and gelfoam (white arrows)

**Fig. 2:**
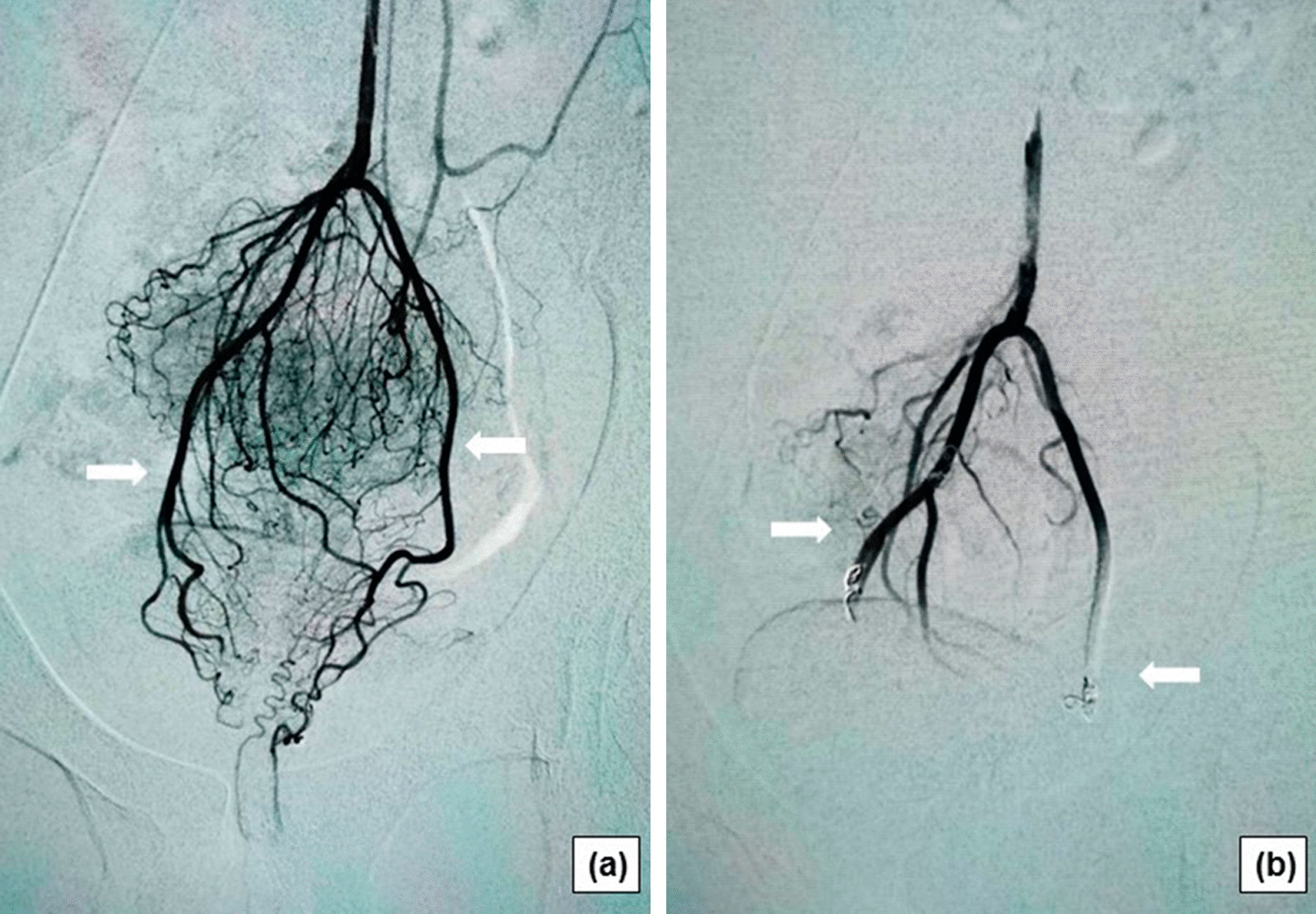
Angiographic features of hemorrhoid artery in patients with internal hemorrhoids. **a** Subtracted arteriogram showing the rectal hypervascularization (white arrows); **b** The final arteriogram showing no visible vascularization of the inferior part of the rectum after embolization of both SRAs using coils and microparticles (white arrows)

After local anesthesia, a modified Seldinger method was used to puncture the right femoral artery, and then a 4-Fr introducer sheath was inserted. Then, the inferior mesenteric artery was catheterized using a 4-Fr Simmons catheter (Cordis, Miami Lakes, Florida), and the SRA and its branches were superselective angiography using a 2.7-Fr microcatheter (Terumo, Tokyo, Japan) (Figs. 1a, 2a). A moderate amounts of gelfoam particles (350–560 μm, Alicon, Hangzhou, China) or microparticles (300–500 μm, Hengrui Medical, Suzhou, China) were injected into the target arteries, and then, several 2–3-mm metallic coils (Cook Nester, Bloomington, IN, USA) were used. The total dose of embolic agents and the number of coils were determined by consensus of two interventional radiologists. The embolization procedure was terminated when the arterial vascular bed in the hemorrhoidal plexus disappeared (Figs. 1b, 2b).

The introducer sheath was pulled out after the operation, and the puncture point was compressed to staunch bleeding and then bandaged with pressure. Intraoperative complications, postoperative symptoms (pain, fever, haemorrhage and tenesmus) were prospectively documented in the electronic medical record. Ischemic complications were also classified according to the reporting standards of the Society of Interventional Radiology (SIR) [16].

Technical success was defined as achievement of complete occlusion of all visible branches of the SRA. The primary endpoint for clinical success was defined as improvements in clinical performance scores (by at least two points for the French bleeding severity score, “Appendix”) [15] for at least six month after SRAE and with no complications. Pain was measured preoperatively, on the day of operation and 2 days postsurgery using the Visual Analogue Scale (VAS) pain score. Clinical follow-up was performed daily until day 3 after embolization or until hospital discharge.

All patients got follow-up by clinic visits or by phone at month 1, month 3, month 6 after discharge, and then every 3 months until 1 year, including evaluation of the clinical conditions and changes in symptoms. Additionally, major and minor complications were also assessed during the follow-up.

### Statistical analysis

Electronic medical records were thoroughly reviewed to collect patient characteristics and details of the embolization procedure. Continuous variables were described by mean and standard deviation, and categorical variables were described by count and percentage. Paired *t* tests or Wilcoxon matched-pairs signed-rank tests were used for continuous variables, and McNemar’s Chi-squared tests were used for discrete variables, for between-group comparisons. Signed-rank tests were used to compare pre- and post-operative clinical scores within groups and score changes between groups. Statistical significance was defined as *p* < 0.05. SPSS version 23.0 software (IBM, Armonk, NY, USA) was used for all analyses.

## Results

### Baseline data

There were 41 patients underwent SRAE, (27 males and 14 females, with a mean age of 47 ± 12 years, range: 25–72 years), 23 with gelfoam particles + coils while 18 with microparticles + coils from March 2017 to June 2020. Depending on the Goligher haemorrhoids classification [14], 16 (39%) patients with grade II internal haemorrhoidal and 25 (61%) patients with grade III (Table 1). The baseline data of the hemorrhoids severity with no significant differences according to the French bleeding severity score (*p* = 0.435).

**Table 1:** Patients characteristics (n = 41)

Characteristics	Value
Age (years)	47 ± 12
*Sex*	
Male	27 (66%)
Female	14 (34%)
*Stage of prolapse (Goligher)*	
Grade II	16 (39%)
Grade III	25 (61%)

### Efficacy, complications and follow-up

Technical success was 100%. The operating time was (49 ± 10) minutes, and there were no immediate or postoperative complications such as intestinal necrosis and infection, femoral arterial puncture site bleeding, hematoma and pseudo-aneurysm. Clinical success did not significantly differ at the initial six months of follow-up between the two groups: 87.0% (20/23) for the Group A vs. 88.9% (16/18) for the Group B (p = 0.098). The post-operative French bleeding severity score was significantly lower than the pre-operative bleeding severity score in each group (p < 0.01, respectively) (Table 2).

**Table 2:** Change in French bleeding severity scores

	Coils + gelfoam particles				Coils + microparticles			
	Before	After	Change	*P* value	Before	After	Change	*P* value
Scores	6 [5–8]	2 [0–5]	− 4 [− 6; − 1]	< 0.01	6 [5–8]	3 [0–6]	− 3 [− 7; − 1]	< 0.01

After six months, six patients (16.7%; 4 in Group A, 2 in Group B) had rebleeding. The 5 patients who failed haemostasis and 6 patients who rebleeding underwent either PPH (n = 5), rubber band ligation (n = 3), inferior rectal artery embolization (n = 2) or sclerotherapy injection (n = 1) during the follow-up period of 7–12 months after conservative treatment failed.

Of all the patients, 34.1 per cent had postoperative pain compared to the pre-surgical pain-free situation. Postoperative VAS scores were between 1 and 6 (mean 2.02 ± 2.20) on the day of operation, and between 0 and 3 (mean 0.37 ± 0.70) on the second day after surgery. Postoperative pain resolved spontaneously without painkillers, and did not affect the time to return to previous daily activities and the length of hospital stay. 48.8 per cent patients (20/41) experienced varying degrees of tenesmus postoperatively which subsided on its own 3 to 7 days later.

## Discussion

This study, similar to findings in other studies [7, 9, 11–13, 15, 17, 18], shows that the SRAE was feasible and safe, and the preliminary haemostatic efficacy had achieved a rapidly clinical symptoms improvement in 36 patients (87.8%) and without major complications.

In recent years, researchers have proposed many hypotheses or theories about the pathogenesis of HD, including its vascular characteristics, that hemorrhoids are believed to be fibrovascular cushions containing arteriovenous communications (or CCR). The CCR is supplied by the SRA, and SRA is basically participated in the pathogenesis of hemorrhoids [3, 19]. Hyperplasia of the arteriovenous network within the anorectal submucosa (or CCR) leads to an increased vascular pressure within hemorrhoids [20].

Many researchers have carried out a series of endovascular treatment and satisfactory results were obtained [7–9, 11–13, 15, 17], based on the vascular nature and anatomy of hemorrhoids proposed by Aigner et al. (transmural branches of the SRA play a crucial role in the arterial blood supply of the CCR) [19]. The arterial flow reduction seems to decrease the hypertension of the CCR [21, 22].

The key point to successful endovascular treatment is to block the feeding artery of hemorrhoids. Therefore, the precise superselection of internal hemorrhoid artery, the optimal embolization end-point and the selection of optimal embolic materials are particularly important. Recently, internal hemorrhoids with embolization or SRAE has been attempted using different embolic materials in prior studies included N-butyl cyanoacrylate (NBCA), polyvinyl alcohol (PVA) particles, gelfoam particles, microspheres and coils, they have similar clinical efficacy and there were no major complications such as ischemic necrosis [5, 7, 11, 23]. In addition, other permanent liquids agents such as ethylene vinyl alcohol copolymer (EVOH) were also used as an embolic agent that show penetration into the haemorrhoidal plexus and eliminate possible connections from other supply branches potential [24].

Coils have the advantage of precise controllable embolization at the bleeding site, however, they lack the ability to reach deep into the hemorrhoidal vessels bed. On the other hand, it is frequently difficult to completely occlude the hemorrhoidal plexus with coils that leads to consequently poor efficacy [25]. Vidal found that 72% of patients showed an significant improvement in clinical symptoms after SRAE, while others did not, that were embolized with coils only [11, 15]. The potential reason may be related with the presence of variations in the feeding arteries, anastomotic collateral vessels, and recanalization of embolized vasculature [15, 25].

Further, gelfoam particles or PVA particles were used for embolization can result in a favorable clinical outcomes that compared with coils only according to the previous literatures [9, 11]. Zakharchenko found that approximately 90% of the patients had bleeding cessation on 2 days after the operation by using PVA particles (300 μm) to embolize the terminal branches and metallic coils (3–5 mm) to embolize the trunk of the SRA [7].

PVA particles has a certain swellability in blood, and it can be used as permanent embolic agents to embolize the distal haemorrhoidal plexus that prevent the formation of collateral circulation thus reducing the high rates of bleeding recurrence. The characteristics of microparticles used in this study were the same as PVA particles that described previously [7].

Additionally, the rectum has a multiple supply of blood from the inferior mesenteric and internal iliac arteries, and the SRA is the only artery that involved in the vascularization of the rectum along the entire length [26]. Furthermore, there was considerable differences in the distribution of the arterial network in the rectal submucosal region, and extensive collateral flow between the branches of the SRA and the internal iliac artery [27]. And, it is also regarded as the major cause of failure in hemostasis following SRAE [28]. Although some studies have shown that the clinical benefit rate of superior and inferior hemorrhoidal artery embolization is higher than that of SRA embolization alone [17]; however, an theorical increased risk of ischemia must be taken into consideration. Similarly, it has not been found that PVA embolization could lead to intestinal ischemic necrosis in the previous animal experiment and clinical studies [7, 11, 29]. In our patients, there was also no rectal ischemia events observed, although the shortest follow-up period was only six months.

In addition, gelfoam particles that considered as absorbable embolic agents, even if the anastomosis of the middle and inferior rectal arteries could not be compensated, the theoretical risk of rectal ischaemic necrosis was also low. Gelfoam particles has hemostatic activity and can also induce thrombosis in a short amount of time when they block the blood flow of hemorrhoidal plexus. In our previous research, we did observe a higher hemostasis rate in the gelfoam particles + coils (84.4%, [8]) than others using coils alone(72%, [5]), but lower rate than others using PVA particles + coils (92.5%, [7]). In this study, we selected embolic materials of similar diameters (350–560 μm vs. 300–500 μm) with no significant difference in initial hemostatic efficiency.

Different embolic agents were used in this study, coils + gelfoam or microparticles. Choice of the embolic materials is usually selected by preference of the operator, and no guideline or consensus to regulate this process. Therefore, to achieve the best clinical outcome by percutaneous endovascular embolization of SRA, the types and doses of embolic agents should to be further investigated.

There are a number of limitations of the present study. First, this was a multicenter retrospective study of prospectively collected data, there were variations in treatment and evaluation among different hospitals and even between doctors. And second, the sample size was small and the study did not compare the effects of embolic agents with different particle sizes. Finally, it is probably too early to say for sure that hematochezia will recur or not at maxmum 15-month follow-up. All these issues should therefore be studied in a randomized clinical trial and with a longer follow-up period.

## Conclusions

We demonstrated that both gelfoam particles and polyvinyl alcohol microparticles in the endovascular treatment of hemorrhoidal bleeding had similarly good short-term efficacy (87.0% vs 88.9%, p = 0.098) and with no major complications. We believe that the SRAE approach were most favorably considered a viable option in cohort of patients with bleeding hemorrhoids.

## Data Availability

The datasets used or analysed during the current study are available from the corresponding author on reasonable request.

## Appendix

See Table 3.

**Table 3:** The French bleeding severity score (maximum worst since treatment 9)

Frequency	Never	0
	≥ 1 per year	1
	≥ 1 per month	2
	≥ 1 per week	3
	≥ 1 per day or per saddle	4
Bleeding	Never	0
	At wiping	1
	In the toilet	2
	In underwear	3
Anemia	Never	0
	Without transfusion	1
	With transfusion	2

## Abbreviations

SRAE: Superior rectal arterial embolization
HD: Hemorrhoidal disease
DG-HAL: Doppler-guided haemorrhoid artery ligation
SRA: Superior hemorrhoidal artery
CCR: Corpus cavernosum recti
SIR: Society of interventional radiology
VAS: Visual analogue scale
NBCA: N-butyl cyanoacrylate
PVA: Polyvinyl alcohol
EVOH: Ethylene vinyl alcohol copolymer
